# Why be moral? Children's explicit motives for prosocial-moral action

**DOI:** 10.3389/fpsyg.2015.00552

**Published:** 2015-05-06

**Authors:** Sonia Sengsavang, Kayleen Willemsen, Tobias Krettenauer

**Affiliations:** Department of Psychology, Wilfrid Laurier University, WaterlooON, Canada

**Keywords:** moral motivation, moral development, autonomy, childhood, prosocial behavior, antisocial behavior, parenting

## Abstract

Recent research on young children's morality has stressed the autonomous and internal nature of children's moral motivation. However, this research has mostly focused on implicit moral motives, whereas children's explicit motives have not been investigated directly. This study examined children's explicit motives for why they want to engage in prosocial actions and avoid antisocial behavior. A total of 195 children aged 4–12 years were interviewed about their motives for everyday prosocial-moral actions, as well as reported on their relationship with their parents. Children's explicit motives to abstain from antisocial behavior were found to be more external and less other-oriented than their motives for prosocial action. Motives that reflected higher levels of internal motivation became more frequent with age. Moreover, positive parent-child relationships predicted more other-oriented motives and greater explication of moral motives. Overall, the study provides evidence that children's explicit moral motivation is far more heterogeneous than prominent theories of moral development (past and present) suggest.

## Introduction

In contemporary research on moral development, it is commonly understood that young children do not engage in prosocial-moral actions out of obedience to authority, fear of punishment, or because of tangible rewards they expect from others, as Piaget (1932/1999) and Kohlberg (1976) would have stressed. Rather, children spontaneously engage in prosocial and moral actions because they genuinely care about others' well-being and because they want to do what they consider right. Many studies seem to converge toward this point. In the following, three prominent lines of research are taken as examples: social domain theory, infants' helping behavior, and development of children's empathy.

Social domain theory has extensively examined children's judgments and evaluations of prototypical moral and conventional transgressions (for an overview, see Smetana, 2006) with the general conclusion that even 3–4 year-olds differentiate between moral, conventional, and prudential concerns, with moral rule transgressions ranked most severely. Whereas, conventional rules are needed in order to maintain societal order, moral rules serve to establish fairness, avoid harm-doing, and promote other's well-being. As domain theory maintains, the obligatory nature of children's morality does not depend on authorities, laws, or customs. Children are motivated to follow moral rules “because it is the right thing to do,” as Nucci (2002) aptly put it. Thus, according to domain theory, moral rules are internally binding to children.

Recent research on children's helping behavior, although starting from largely different conceptual and methodological grounds, arrives at similar conclusions. In a series of experiments, Warneken and Tomasello (2006, 2007) demonstrated that 14–18 months-old infants spontaneously engage in helping behavior without being explicitly asked to do so and without being offered a benefit in return. Furthermore, it was found that offering extrinsic rewards for helping behavior undermined children's motivation to help others (Warneken and Tomasello, 2008), whereas the presence vs. absence of parents did not influence the frequency of children's helping (Warneken and Tomasello, 2013). From these findings, the authors conclude that young children are intrinsically motivated to help others regardless of instrumental rewards, and regardless of any desire to please their parents. It is suggested that this intrinsic motivation to help others forms an important evolutionary basis for human altruism (Warneken and Tomasello, 2009).

Instrumental helping as studied by Warneken and Tomasello is an early form of prosocial behavior that does not imply a profound understanding of other's emotional needs, nor does it require the reconciliation of prosocial concerns with egoistic desires (cf. Dunfield et al., 2011). Even though more demanding forms of prosocial behavior, such as comforting and sharing with others, typically need more time to develop (Svetlova et al., 2010), they seem to be no less internally motivated. A vast body on children's empathy development demonstrates that prosocial behavior is strongly motivated by children's ability to respond empathically to others' needs (Eisenberg et al., 2006). By contrast, personal distress in reaction to others' emotions potentially undermines prosocial behavior (Eisenberg et al., 2010). Many studies demonstrated that children's sensitivity to others' needs grows in the context of positive and supportive relationships with caregivers (e.g., Thompson et al., 2006). Thus, whereas Warneken and Tomasello (2006) emphasize the evolutionary nature of instrumental helping, this by no means implies that social factors are unimportant for prosocial development (cf. Pettygrove et al., 2013; Hammond and Carpendale, 2015).

In summarizing these and related findings from other research areas (e.g., theory of mind, attachment), Thompson (2012) in a paper entitled “Whither the preconventional child?” suggested to abandon the notions of moral heteronomy and preconventional morality altogether that were typically used to characterize childhood. According to Thompson, these notions obstruct progress toward a life span theory of moral development as they distort our understanding of the developmental preconditions for moral maturity. “As researchers of early moral development have appreciated, the conceptual skills of the early years are a foundation for the development of a humanistic, cooperative, and relational moral orientation, not an obstacle to be overcome in later years” (Thompson, 2012, p. 426).

Naturally, research on early moral development is based on behavioral observations and thus focuses on children's implicit motives for prosocial-moral action. However, any theory of moral development has to deal with the fact that morality is not limited to the level of implicit motives. It is an important developmental achievement prompted by growing cognitive and verbal abilities that children become increasingly aware of their emotions, desires, and goals (see, for example, Flavell, 1999). Moral development is no exception from this general trend. Children develop explicit conceptions about their moral preferences (Krettenauer et al., 2013). These explicit preferences do not necessarily match with implicit motives.

It is generally assumed that implicit and explicit motives follow different developmental trajectories (McClelland et al., 1989). Whereas, implicit motives are shaped early in life by preverbal and affective experiences, explicit motives are assumed to develop through verbally mediated learning. Moreover, the two forms of motivation tend to influence different types of behavior. Implicit motives tend to predict spontaneous behavior over extended periods of time. By contrast, explicit motives were found to be more predictive of decision-making in situations that are of perceived relevance to these motives. As moral action manifests itself in spontaneous behaviors as well as deliberate decision-making, any account of children's moral motivation that is solely based on implicit motives appears to be incomplete. Thus, whereas research provided strong evidence that children's prosocial-moral behavior is internally motivated on the implicit level, it has to be considered an open question whether this internal motivation is present on the level of explicit motivation, as well. This question defines the focus of the present paper.

In addressing the issue of children's explicit motives for prosocial-moral action, we do not intend to question previous research on infants' implicit prosocial motivation, nor do we claim that children's explicit motives for prosocial-moral action to date have been a completely uncharted territory of research on moral development (for a discussion of previous research and its limitations see section below). Rather, we want to draw the readers' attention to the fact that any appreciation of children's morality that is solely based on implicit motives seems to be incomplete. As children develop explicit motives for prosocial-moral action, a second motivational system emerges that is partly independent (but certainly not fully separated) from children's implicit motivation. Piaget (1932/1999) maintained that moral development in action precedes the forms of heteronomous and autonomous morality he observed at later developmental periods (cf. Carpendale, 2009; Hammond, 2014). Thus, the intrinsic nature of moral motivation found in young children may not be a dominant feature throughout the course of development. Following this perspective, it may be premature to fully abandon the founding theories of research on moral development (Piaget and Kohlberg) as Thompson (2012) suggested. Rather, it might be advisable to preserve those insights of Piaget's and Kohlberg's theories that seem to be valid today and to discard only those aspects that were misleading.

Research on children's social-moral reasoning has a long history in developmental psychology but is of limited value when it comes to investigating explicit motives for moral actions, for various reasons. As described before, social domain theory offers an internalist view of children's moral motivation by stressing the autonomous nature of young children's justifications of moral rules. However, children's cognitive understanding of moral rules does not necessarily correspond with their level of moral motivation (Nunner-Winkler, 2009). Justifications for why people in general should (not) engage in certain behaviors do not automatically provide the corresponding motives for moral action, as various models that link moral judgment with action imply (for an overview, see Garz et al., 1999). From this perspective, rule justification are not a valid indicator of explicit motives, whereas research that asks children to make a deliberate decision in a given situation and to provide reasons for this decision is closer to tapping into explicit moral motives. However, past research on children's moral decision-making was mostly based on Kohlberg's theory and methodology, using dilemma situations that were highly remote to children as well as a concept of moral stages that largely ignored the variability of reasoning within and across situations.

A notable exception is Eisenberg's (1986) research on the development of children's prosocial reasoning. Eisenberg used a broad range of categories for coding reasons children provided when making prosocial decisions. In line with research on children's implicit motives, Eisenberg found that concern for others' needs played a pivotal role for prosocial decisions. At the same time, however, egoistic self-concerns (including concerns about direct reciprocity similar to Stage 2 reasoning in Kohlberg's scheme) were not uncommon, especially in younger children (for similar findings, see Keller, 1996). Moreover, the use of internalized affect (positive as well as negative) as a reason for prosocial choices significantly increased with age. Overall, young children's explicit motives were less intrinsic than research focusing on implicit motives suggests.

However, in Eisenberg's (1986) studies, only prosocial dilemmas were useds in which the role of prohibitions, authorities, and formal obligations was deliberately minimized. This defines an important limitation of this research, as morality certainly goes beyond prosocial actions of helping, sharing, or caring. Morality includes both prescriptions (do's) and proscriptions (don'ts). Prescriptions or positive moral obligations focus on promoting others' well-being, whereas proscriptive or negative morality focuses on harm avoidance. The difference between positive and negative morality has important implications for self-regulation, motivation, and emotions (Janoff-Bulman et al., 2009; Sheikh and Janoff-Bulman, 2010; Krettenauer and Jia, 2013). As Kochanska (2002) demonstrated, the two demand contexts evidence distinct links with rule internalization. Correspondingly, children's explicit motives for prosocial actions might differ from the motives that prevent them from intentionally harming others.

### The present study

The present study aimed at investigating children's explicit motives for prosocial-moral actions while avoiding the shortcomings of past research outlined above. Thus, children's moral motives were not inferred from rule justifications or decision-making in hypothetical dilemma situations. Instead, children were explicitly asked what motivates them to engage in everyday prosocial-moral actions, such as sharing and helping, as well as not stealing or not pushing others. These everyday moral actions were systematically sampled from the two contexts of prescriptive and proscriptive morality in order to avoid any overgeneralization by drawing from one behavioral context alone. Thus, particular attention was devoted to the context-specificity of children's explicit moral motives.

Three questions were addressed: (1) What are important explicit motives for children's prosocial and moral actions? (2) How do these motives change with age? (3) In what way are children's explicit motives for prosocial-moral action related to parenting?

In line with findings reported by Eisenberg (1986), it was expected that concerns for others' needs are an important explicit motive for prosocial actions, but less so in the context of negative morality. In order to prevent antisocial behavior among children, parents often provide verbal directives and enforce rule compliance (e.g., Dahl and Campos, 2013). As a consequence, children's avoidance of antisocial behavior may be more externally motivated than their prosocial actions. Regardless of these context differences, it can be assumed that children in general become better able to express their motives for prosocial-moral actions with increasing age due to growing verbal and cognitive abilities. Thus, a general increase in children's ability to articulate their motives for prosocial-moral actions was expected. Finally, it was expected that positive parenting, which is supportive of children's autonomy, would be associated with higher levels of internal moral motivation as reflected in children's explicit motives. As was demonstrated before, social contexts that are supportive of self-determination generally lead to an increased congruence between explicit and implicit motives (Thrash et al., 2010; Schattke et al., 2011). As a consequence, children who experience the relationships with their parents as harmonious and supportive may be better able to articulate their implicit motives for prosocial-moral action.

## Methods

### Participants

The sample consisted of 195 children from Junior- and Senior-Kindergarten (JK-SK; *n* = 56; *M* = 5.56 years; *SD* = 0.59; 35 males), Grades 2–3 (*n* = 70; *M* = 8.49 years; *SD* = 0.58; 35 males), and Grades 5–6 (*n* = 69; *M* = 11.46 years; *SD* = 0.59; 36 males). The participants ranged in age from 4 to 12 years (*M* = 8.65; *SD* = 2.44; 51.3% males). Information letters and consent forms were sent home with children from consenting elementary schools in Kitchener/Waterloo, Ontario, Canada. The schools were located in socially heterogeneous neighborhoods with predominantly middle-class background. Parental written consent and children's oral assent were obtained before participants were asked to do an interview and complete a questionnaire. For participating in this study, children in Kindergarten received a small gift from an assortment of choices worth approximately $5 (e.g., puzzles, toy cars, skipping ropes). Children in Grades 2–3 were given a choice of a small toy or $5 in cash, and Grades 5–6 received $5 in cash. The Research Ethics Board at Wilfrid Laurier University approved this study.

### Measures and procedure

Mixed-methods were employed including questionnaires and individual child interviews. All interviews were audio taped and transcribed verbatim. The average length of time to complete the entire interview and questionnaire was approximately 30–40 min.

#### Explicit motives for prosocial-moral action

Participants completed the *Children's Moral Self Puppet Interview* (Krettenauer et al., 2013; Sengsavang and Krettenauer, 2015). In this interview, children watched 23 short videos of two puppets engaging in a dialog about (im) moral preferences. One puppet states a preference for moral behavior or, alternatively, an aversion to immoral behavior, whereas the other puppet states the opposite (e.g., “I like to be kind to others” vs. “I don't like to be kind to others”; “I like to make other children angry” vs. “I don't like to make other children angry”). Children are asked to choose between one of the two puppets. In 10 of the 23 videos, children are further asked *why* they would want to engage in the described prosocial behavior or why they would not want to act in this antisocial way (e.g., “Why would you want to be kind to others?,” “Why would you not want to make other children angry?”). These ten interview questions were used to assess children's explicit motives for prosocial-moral actions. Five questions described everyday prosocial behaviors of helping, sharing and caring for others, and five questions addressed antisocial behaviors of physical and verbal aggression, as well as stealing (for a full list of the interview questions see Appendix). The ordering of the questions was randomized in the interview.

#### Coding

Coding categories were derived from approximately 40 randomly chosen transcripts. Categories were meant to reflect children's motives for prosocial-moral actions on a continuum from external to internal as described by Self-Determination Theory (Ryan and Deci, 2000). Moreover, categories were defined in order to capture common themes of prosocial-moral reasoning as reflected in previously established coding systems (e.g., Eisenberg, 1986; Colby and Kohlberg, 1987; Gibbs et al., 1992; Keller, 1996). External motives for prosocial moral actions were present when children referred to *Standards and rules* as motives for moral actions including global act evaluations (e.g., “Because my mom and dad told me not to do it”, “Because, I should not do this,” “Because it is not nice”). By contrast, the most internal form of moral motivation was present when children referred to their own *Personal-moral preferences* including emotions as a motive for prosocial-moral action (e.g., “Because I don't want others to be hurt,” “Because it makes me feel good inside to help”). Between these two polar extremes were *Self-interested, Other-oriented* and *Fairness-related* motives. *Self-interested* motives were evident when a child referred to positive or negative consequences to the self as a motive for prosocial-moral action (e.g., “Because then they could hurt me,” “If I am not kind, I will not have any friends”). *Other-oriented* motives appeared when a child referred to others' needs and feelings, or the consequences of an action for others (e.g., “Because it might hurt their feelings,” “So that they have something to play with”). In *Fairness-related* responses, children elaborated on the rightness or wrongness of an action by taking another person's perspective and balancing conflicting interests (e.g., “I would not want others to hurt me,” “Because it's not fair for everybody, and everybody should be treated the same way”). In addition, there appeared responses that were either *Unelaborated* or *Unscorable*. Responses were considered *Unelaborated* when children did not give a qualified answer that went beyond what was implied in the interviewer question (e.g., “Just because,” [Why do you want to help another child who is hurt?] “Because I want to help”). Finally, responses were considered *Unscorable* when a qualified response was given but its meaning remained too vague or too idiosyncratic in order to fit into one of the categories described above. Using a subset of 50 randomly chosen interviews, two independent raters obtained 92% agreement for the coding system, κ = 0.90.

If a child articulated more than one motive for why s/he would want to engage in prosocial-moral actions, all motives were coded separately. Thus, multiple codings were possible. This reduced linear dependency between codes. Overall, 2382 codes were generated. Of these coded responses, 18.5% referred to *Standards and rules*, 9% were *Self-interested*, 38% *Other-oriented*, 4.6% *Fairness-related*, and 9.3% referred to *Personal-moral preferences*. Moreover, 8.1% of responses were *Unelaborated* and 12.4% were considered *Unscorable*.

For all further statistical analyses, motivation category scores were tallied across the five prosocial and the five antisocial interview questions separately, yielding scales that represent how often each category was used in the two contexts (range 0–5; for *M*s and *SD*s see Table 1).

**Table 1:** **Means and SDs for explicit moral motives in antisocial and prosocial contexts**.

	**Antisocial**		**Prosocial**	
	***M***	***SD***	***M***	***SD***
Unelaborated	0.36^a^	1.01	0.33^a^	0.99
Standards and rules	1.06^a^	1.24	0.55^b^	0.94
Self-interested	1.37^a^	1.32	0.37^b^	0.72
Other-oriented	1.26^a^	1.23	2.05^b^	1.64
Fairness-related	0.52^a^	0.85	0.42^a^	0.98
Personal-moral	0.36^a^	0.63	0.45^a^	0.80

*N = 193*.

a,b *Means in the same row with different superscripts are significantly different (t-test, p <0.008)*.

#### Parent-child relationships

Participants completed the *Network of Relationships Inventory* (NRI; Furman and Buhrmester, 1985) to assess a broad range of relationship qualities. This measure has been used extensively in previous research with children ranging from 6 to 13 years old (e.g., Field et al., 2007; Rubin et al., 2004). The 13-item short form of the NRI was used in the current study. The short-form includes two factors: seven items representing *support* (e.g., “How much do you share your secrets and private feelings with your mother”) and six *negative interaction* items (e.g., “How much do you and your mother disagree or quarrel with each other?”). Participants answered the same set of questions for their relationship with a mother figure and then again with a father figure. Participants rated the extent each individual satisfies every item based on a four-point scale ranging from (1) little or none to (4) extremely much. For the 4–6-year-olds, the interviewer read the questions from the NPI aloud, whereas the 8–9-year-olds were given the option to complete the questionnaire privately or together with the interviewer. The oldest age group completed the questionnaire themselves.

In the current sample, there were significant and positive associations between both maternal and paternal support (*r* = 0.64, *p* < 0.01), as well as maternal and paternal negative interaction (*r* = 0.57, *p* < 0.01). Accordingly, summary variables were created to represent *parental support* and *parent-child negative interaction*. Internal consistency was high for both the parental support (α = 0.82) and negative interaction (α = 0.88) aggregate variables.

## Results

### Effects of context and age

In order to investigate whether children's explicit motives for prosocial-moral action vary by age and context, a Three-Way mixed model multivariate analysis of variance (MANOVA) was performed: age group (kindergarten, grades 2–3, grades 5–6) by context (antisocial, prosocial) by category (unelaborated, standards and rules, self-interested, other-oriented, fairness-related, personal-moral). Note that the unscorable coding category was not used in all further analyses as it was of limited theoretical interest.

The MANOVA revealed no significant main effect of age group and significant main effects of context as well as category (see Table 2). The two main effects were qualified by two significant Two-Way interactions: (a) an interaction between context and category and (b) an interaction between age group and category. Thus, children's explicit moral motives varied by context (prosocial vs. antisocial), as well as by age.

**Table 2:** **Moral motives by age group, context and category: results of mixed model MANOVA**.

	**df**	***F***	**η^2^_p_**
Age group	2, 190	1.96	0.02
Context	1, 190	49.83^***^	0.21
Category	5, 186	36.95^***^	0.50
Age group × context	2, 190	1.87	0.01
Age group × category	10, 374	8.61^***^	0.19
Context × category	5, 186	33.35^***^	0.47
Age group × context × category	10, 374	1.84	0.05

*All F-values reported are based on Pillai's Trace test statistic as it is considered most robust. Other test statistics (Wilk's Lambda, Hotelling's Trace, Roy's Largest Root) yielded slightly different F-values for some interactions, but all p-values reached the same level of significance*.

*** *p < 0.001*.

Mean differences of children's moral motives by context are summarized in Table 1. Three motives were most salient in the antisocial context: standards and rules (*M* = 1.06, *SD* = 1.24), self-interested (*M* = 1.37, *SD* = 1.32), and other-oriented (*M* = 1.26, *SD* = 1.23). For the prosocial context, by contrast, the other-oriented category was by far the most prominent (*M* = 2.05, *SD* = 1.64). Pairwise comparisons (*t*-test, *p* < 0.05 Bonferroni-corrected) yielded significant differences between the prosocial and antisocial context for standards and rules, self-interested, as well as other-oriented motives. Thus, children referred to standards and rules, as well as self-interest more often in the antisocial context, whereas other-oriented motives were more salient in the prosocial context.

The significant interaction between age group and category was followed-up by a *post-hoc* discriminant descriptive analysis (DDA; see Warne, 2014) to determine the function that distinguishes the age groups from each other on the category scores. Two discriminant functions were created given that the number of functions is equal to *k* (groups)—1. The first function was statistically significant (*p* < 0.001), while the second was not (*p* = 0.07). Thus, the second discriminant function was not further considered. The evaluation of the standardized discriminant coefficients for the first function revealed that fairness had the strongest effect (0.715), followed by unelaborated (−0.506), personal-moral (0.335), other-oriented (0.246), self-interested (−0.200), and standards and rules (−0.012).

Parallel discriminant ratio coefficients (DRC; Thomas, 1992) were calculated to determine variable importance. For this function, the strongest parallel DRCs were found for fairness-related (0.498), unelaborated (0.296), and for personal-moral preferences (0.130). These parallel DRCs indicate that these three categories were the more important variables for distinguishing between age groups. Overall, unelaborated responses decreased with age, whereas reference to fairness-related motives and personal-moral preferences increased (see Table 3).

**Table 3:** **Means and SDs for moral motives by age group**.

	**Kindergarten**		**Grade 2-3**		**Grade 5-6**	
	***M***	***SD***	***M***	***SD***	***M***	***SD***
Unelaborated	0.94	1.49	0.11	0.34	0.12	0.38
Standards and rules	0.82	0.94	0.97	1.11	0.62	0.62
Self-interested	0.94	0.90	0.97	0.96	0.70	0.78
Other-oriented	1.43	1.25	1.75	1.25	1.74	1.11
Fairness-related	0.02	0.09	0.44	0.81	0.87	0.81
Personal-moral	0.17	0.38	0.43	0.57	0.57	0.72

*N = 193*.

### Effects of parent-child relationship

Regression models were performed for each motive category to examine how children's explicit motives for prosocial- moral action are related to supportive parenting. For this purpose, scales were combined across the prosocial and the antisocial context. Both gender and exact age were entered in Step 1 of the regression models to control for these potentially confounding effects. Parental support and parent-child negative interaction were entered in Step 2. Parent-child relationship variables significantly predicted two moral motivation categories after controlling for effects of age and gender: unelaborated and other-oriented (see Table 4). Parental support negatively predicted children's unelaborated moral motivation, whereas the reverse effect was obtained for parent-child negative interaction. For other-oriented moral motivation, negative parent-child interaction turned out to be a significant predictor. Lower levels of parent-child negative interaction were associated with more other-oriented moral motivation.

**Table 4:** **Results of regression analyses predicting unelaborated and other-oriented moral motivation**.

	**Unelaborated**				**Other-oriented**			
	**Step 1**		**Step 2**		**Step 1**		**Step 2**	
	**β**	***t***	**β**	***t***	**β**	***t***	**β**	***t***
Gender	−0.03	−0.41	−0.03	−0.41	0.10	1.34	0.05	0.72
Age	−0.29	−4.14^**^	−0.29	−4.14^**^	0.08	1.14	0.08	1.17
Parental support			−0.15	−2.15^*^			0.10	1.36
Parent-child negative interaction			0.15	2.13^*^			−0.21	−2.97^**^
Δ*R*^2^	0.09^**^		0.05^**^		0.02		0.06^**^	

*N = 190*.

** p < 0.01,

* *p < 0.05*.

## Discussion

The present study was meant to investigate children's explicit motives for why they want to engage in prosocial actions and do not want to behave antisocially. Contrary to Piaget (1932/1999) and Kohlberg (1976), recent research stressed the autonomous and internal nature of children's moral motivation. However, this research has been mostly focused on children's implicit motives for prosocial-moral actions, whereas their explicit motives have not been directly investigated so far.

In line with previous research, it was found that other-oriented motives, overall, were most salient in children's explicit responses to the question why they want to engage in prosocial-moral actions. Other-oriented motives were already dominant in 4–6-year-olds and did not significantly increase with age. Thus, a concern for others' feelings and needs clearly plays an important role in children's explicit moral motivation. However, this general finding needs to be qualified in various respects.

Other-oriented motives were particularly salient in the context of prosocial actions. In contradistinction, in the antisocial context children referred equally often to external standards and rules (e.g., “I should not do this”), as well as to self-interest (e.g., “They could hurt me”). According to Kohlberg's scheme, these responses would qualify as *preconventional* (Stage 1 and Stage 2). Whereas, Kohlberg's theory would predict a decrease in these responses, in the present study no significant effects of age were found. External standards and self-interest were important concerns even for 10–12-year-olds. Thus, children's explicit motivation to abstain from antisocial or aggressive behavior appears to be less autonomous and less internal than current research on children's morality suggests.

Whereas, reference to standards and rules as well as self-interest did not change with age, age-related differences in children's explicit motives were found for other categories. First, unelaborated responses declined between the age of Kindergarten and Grade 2–3. Thus, with increasing age, children became better able to explicate their motives for prosocial-moral action. This finding is reminiscent of the Piagetian notion that children's moral development starts from morality in action and later becomes cognitively (re)constructed (cf. Hammond, 2014).

Secondly, it was found that fairness-related (e.g., “I would not want others to hurt me”) and personal-moral preferences (e.g., “I don't want others to be hurt”) responses increased with age. Both types of responses indicate higher levels of organismic integration as described by Self-Determination Theory (Deci and Ryan, 2014). Thus, even though children's implicit moral motivation from an early age can be characterized as autonomous, particularly in the context of prosocial actions, this by no means precludes further development with regard to the integration of morality into the self (see Krettenauer, 2014).

As was found in the present study, children who experienced their parents as supportive and who reported fewer negative interactions with their parents were more other-oriented and were better able to explicate their motives for moral action. Both findings are consistent with previous research. It has been demonstrated repeatedly that children's sensitivity to others' needs grows in the context of positive and supportive relationships with caregivers (e.g., Thompson et al., 2006). At the same time, social contexts that support self-determination led to greater autonomy and congruencies between implicit and explicit motives (Schattke et al., 2011; Deci and Ryan, 2012).

The present study was limited in various respects. First of all, data were cross-sectional. Thus, differences between age groups may be due to factors other than age. Second, the study did not include measures of children's implicit motives for prosocial-moral action. As a consequence, it was not possible to investigate the degree of (in) consistency between these two types of motivation. Third, measures of parent-child relationship were based on children's self-report and may not accurately reflect actual parent-child interactions. Finally, as with all explicit measures, the study's assessment of children's explicit motives for prosocial-moral actions may be susceptible to various self-presentation and social desirability response biases. Even though a measure of social desirability response bias that was part of the *Children's Moral Self Puppet Interview* (for details see Krettenauer et al., 2013) did not yield significant associations with any moral motive category (correlations ranged between *r* = −0.11 and 0.10, *p'*s > 0.10), it cannot be ruled out that children responses were influenced by demand characteristics of the interview situation. Please note, however, that the verbal methods employed in the present study are very well in line with contemporary research designed to elucidate children's moral development (e.g., Malti et al., 2009; Nunner-Winkler, 2009; Weller and Lagattuta, 2014).

Regardless of these limitations, the present study suggests that it may be premature to fully abandon Piaget's and Kohlberg's theories. To be sure, both theories do not adequately grasp the moral autonomy that is evident particularly in young children's implicit moral motivation. However, there are heteronomous elements in children's morality, notably when children provide explicit reasons for why they do not want to engage in antisocial or aggressive behaviors. These heteronomous elements likely reflect the social fact that antisocial behavior is often actively prohibited by parental authorities and sanctioned as a vast body of research on discipline encounters suggests (cf. Grusec, 2006). Thus, this finding aligns well with Piaget's perspective on moral development, where moral heteronomy is attributable to imbalances in power relationships (see Helwig, 2008). Whereas, Piaget and Kohlberg both stressed the heteronomous nature of children's morality, current research emphasizes its intrinsic, cooperative, and autonomous features. Ultimately, it may be misleading to assume a homogenous motivation from which all moral action develops. Moral motivation in childhood and beyond is multi-faceted and much more heterogeneous than prominent theories on moral development (past and present) suggest.

## Author note

SS, KW, and TK, Department of Psychology, Wilfrid Laurier University, Waterloo, Canada. The research presented in this paper was supported by an Insight Grant of the Social Sciences and Humanities Research Council Canada to the third author (Grant number: 435-2012-1070).

### Conflict of interest statement

The authors declare that the research was conducted in the absence of any commercial or financial relationships that could be construed as a potential conflict of interest.

## Interview questions

### Avoidance of antisocial behavior

1. Why would you not want to push other children?
2. Why would you not want to take something that does not belong to you?
3. Why would you not want to make other children angry?
4. Why would you not want to tease other children?
5. Why would you not want to break other children's toys?

### Preference for prosocial behavior

1. Why would you want to help another child who is hurt?
2. Why would you want to share candies with other children?
3. Why would you want to be kind to others?
4. Why would you want to give away your toys to children who do not have enough to play with?
5. Why would you want to get food for a child you know is hungry?
